# Spatial potential ripples of azimuthal surface modes in topological insulator Bi_2_Te_3_ nanowires

**DOI:** 10.1038/srep19014

**Published:** 2016-01-11

**Authors:** Miguel Muñoz Rojo, Yingjie Zhang, Cristina V. Manzano, Raquel Alvaro, Johannes Gooth, Miquel Salmeron, Marisol Martin-Gonzalez

**Affiliations:** 1 IMM-Instituto de Microelectrónica de Madrid (CNM-CSIC), Isaac Newton 8, PTM, E- 28760, Tres Cantos, Madrid, Spain; 2 Materials Sciences Division, Lawrence Berkeley National Laboratory, 1 Cyclotron Road, Berkeley, CA 94720, United States; 3 Applied Science and Technology Graduate Program, University of California at Berkeley, Berkeley, CA 94720, USA; 4 Institute of Nanostructure and Solid State Physics, Universität Hamburg, Jungiusstrasse 11, 20355 Hamburg, Germany

## Abstract

Topological insulators (TI) nanowires (NW) are an emerging class of structures, promising both novel quantum effects and potential applications in low-power electronics, thermoelectrics and spintronics. However, investigating the electronic states of TI NWs is complicated, due to their small lateral size, especially at room temperature. Here, we perform scanning probe based nanoscale imaging to resolve the local surface potential landscapes of Bi_2_Te_3_ nanowires (NWs) at 300 K. We found equipotential rings around the NWs perimeter that we attribute to azimuthal 1D modes. Along the NW axis, these modes are altered, forming potential ripples in the local density of states, due to intrinsic disturbances. Potential mapping of electrically biased NWs enabled us to accurately determine their conductivity which was found to increase with the decrease of NW diameter, consistent with surface dominated transport. Our results demonstrate that TI NWs can pave the way to both exotic quantum states and novel electronic devices.

Three-dimensional (3D) topological insulators (TIs) are a class of materials with a bandgap in the 3D bulk and exotic two-dimensional (2D) metallic surface states that are protected by time-reversal symmetry and are thus predicted to be immune to inelastic scattering by defects and nonmagnetic impurities^1^,^2^,^3^,^4^. The TI surface states form a Dirac cone, comprising charge carriers that mimic massless Dirac fermions, described by relativistic transport equations. The effective massless charge carriers, combined with the potentially low scattering rate in the TI surface states are expected to result in an extremely high carrier mobility. This generates great opportunities for dissipationless quantum electronics and ultra-fast information processing devices. Recent experiments^5^ on single crystals of Bi_2_Te_3_ and Bi_2_Se_3_ have demonstrated that while Fermi levels far in the cones (inside the bandgap) helicity provides strong preservation of backscattering, close to the Dirac point, defects within the 3D bulk cause random local gating of the 2D TI surface states, resulting in spatially fluctuating charge puddles at the TI surface that act as scattering centers^6^. In the 2D surface states of 3D TIs the bulk defects play a similar role as the substrate impurities in graphene^7^,^8^,^9^. This is particularly relevant for potential TI applications, since tuning the chemical potential into the bulk band gap is one of the most common strategies to suppress parasitic bulk conduction in 3D TIs^6^,^10^,^11^,^12^,^13^,^14^ and thus take full advantage of the surface states. In fact, doped 3D TIs have recently been shown to exhibit a strongly decreased mobility compared to intrinsic samples, originating from mesoscopic structures on the length scale of several tens of nm^6^. Although not on purpose, the remarkable predictions for the electronic properties of 3D TI surface states may also simply suffer from unintentional doping, typical for TI materials like Sb_2_Te_3_, Bi_2_Te_3_ and Bi_2_Se_3_, due to small formation energies of antistructure-type defects^15^,^16^, posing significant challenges for TI applications.

TI NWs represent a promising alternative to bulk 3D TI materials since their enhanced surface-to-volume ratio leads to a strongly suppressed bulk transport channel^17^,^18^,^19^,^20^, not necessarily requiring doping to maximize the ratio of surface to bulk conductance. Another key advantage of TI NWs compared to planar TIs is their unique surface band structure, providing an additional parameter space to realize nanoscale quantum and spintronic devices. Due to the confined periodic boundary conditions around the NWs’ perimeter, the 2D Dirac fermions form discrete 1D subbands^21^,^22^,^23^,^24^. Such 1D azimuthal modes, tailored by magnetic flux quanta, have already been used as Aharonov-Bohm interferometers^21^,^24^ and are expected to realize 1D topological superconducting states immune to disorder^22^, to host 1D spin polarized currents^23^ as well as to manipulate Majorana zero-modes^23^. But just like for planar 3D TIs all calculations so far have been obtained for nanowires with a perfectly equipotential surface. Experimentally investigating the TI surface states in nanowires remains very challenging^25^,^26^, especially at room temperature, in part because the lateral size of these structures is below the resolution of the Angle Resolved Spectroscopy instruments commonly used to investigate the band structure of TI materials^2^,^4^,^27^. Hence, while to date the influence of defects in the bulk on the 2D surface states has been recently investigated, the robustness of surface states of TI nanowires against bulk defects remains an open question. It is not straightforward that the properties of 2D TI surface states or graphene can directly transform into the azimuthal 1D surface state of nanowires, because their electronic structure is remarkably different.

Here we use Kelvin probe microscopy (KPM)^28^,^29^ at room temperature to map the surface potential distribution of individual, suspended Bi_2_Te_3_ NWs with diameters of 45 nm, 75 nm and 250 nm. While no modulation of the surface potential around the NWs’ perimeter is observed, however, the KPM measurements reveal spatial potential ripples along the NWs’ axis that can not be clearly associated to the surface topology of the nanowire. Our results show that the azimuthal 1D modes around TI NWs’ surface are relatively robust against defects and diameter variations, but their spatial distribution along the NWs axis can be significantly altered.

## Device and Measurement Setup

**Figure 1:**
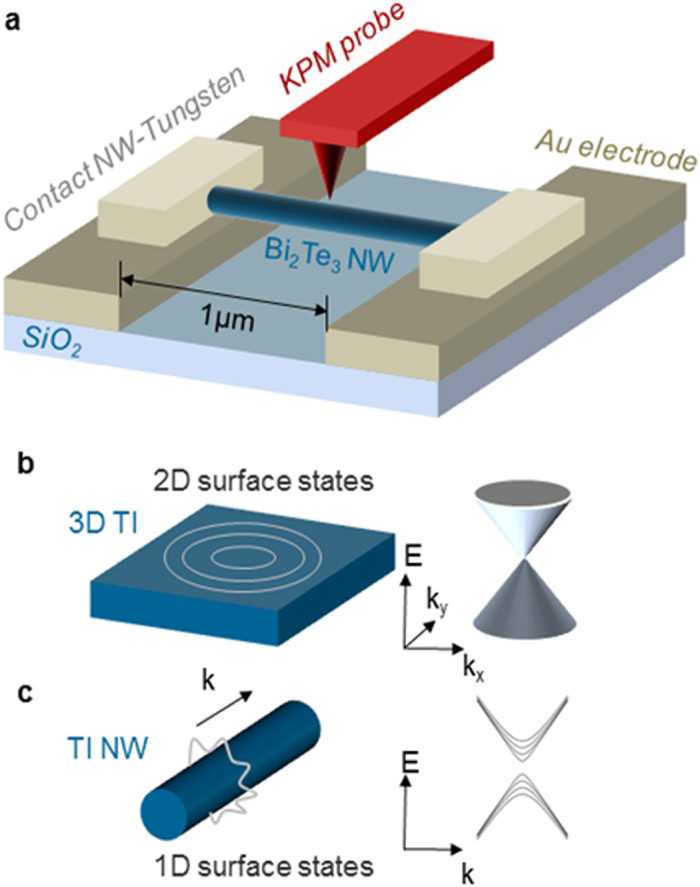
(**a**) Schematic diagram of the measurement set-up. (**b**) 3D topologoical insulators (TIs) exhibit 2D surface states, forming a Dirac cone. (**c**) TI nanowires exhibit 1D subbands on the surface, due to the quantum confined boundary condition along the perimeter direction.

The Bi_2_Te_3_ NWs are grown *via* an electrodeposition technique^30^,^31^(Supporting Information), resulting in n-type structures. To probe the transport properties, two-terminal devices with the NW placed on top of two gold electrodes are produced, separated by ~1 μm gap (Fig. 1). By drop cast, the nanowires were suspended on the gap between electrodes, whose depth is around 200 nm that is equivalent to the thickness of the gold electrodes. Electrical contacts to the NWs were fabricated by removing the surface oxide and depositing tungsten *via* focused ion beam. Tungsten serves as the contact^32^ to the NW while the Au pads (touching the tungsten) are connected to external circuits for biasing the NW. To resolve the surface potential (chemical potential) of the NWs, the completed devices were subsequently transferred into a home-built KPM setup, which measures topography and surface potential distribution simultaneously, with ~20 nm spatial resolution on planar surfaces and ~10 mV potential resolution^28^,^33^, allowing us to decipher the microscopic conduction mechanism of the NWs. On curved protruded surfaces the spatial resolution of our setup is even higher. This was demonstrated by accurately determining the work function of single nanoparticles with a diameter of ~10 nm^33^. This resolution is smaller than the diameter of all the measured NWs. KPM is performed at 300 K under inert nitrogen atmosphere. In this KPM setup higher surface potential corresponds to lower work function, and the absolute value of the surface potential is calibrated by setting that of the grounded Au electrode to zero. We note that the naturally grown 3 nm thick surface oxide on the Bi_2_Te_3_ nanowires does not affect the KPM detection of the TI surface states. Contrary to non-topological surface states, TI surface states have recently been shown to be immune to surface imperfections^34^. Although the presence of oxide may induce a shift in surface potential^35^, this shift should be constant over the whole nanowire surface. Therefore the measured spatial variation of the surface potential accurately represents that of the surface states. It is also worth mentioning that as the KPM is a non-contact technique, the nanowires do not become damaged or distorted by the probe during the measurement. We note that other KPM measurements of topologically trivial nanowires, such as Si NWs^36^, did not show any ripple structures.

## Potential ripples

**Figure 2:**
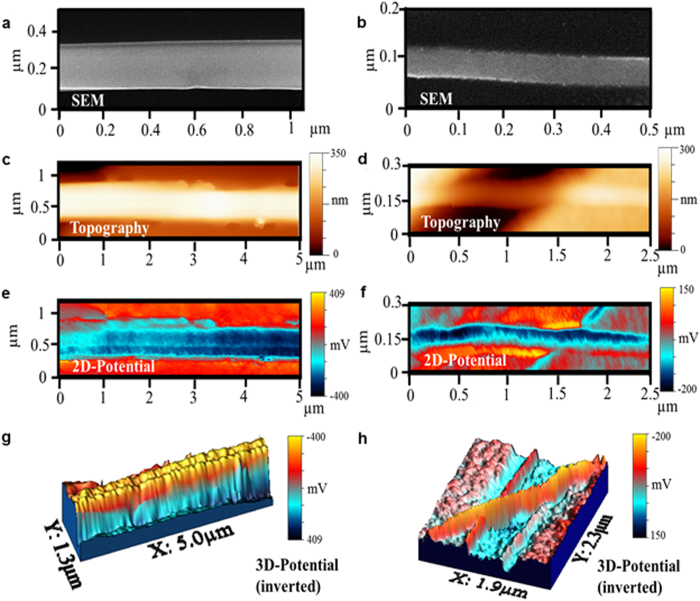
Surface potential ripples of Bi_2_Te_3_ nanowires. SEM, (**a,b**), and AFM topography, (**c,d**), images of 250 nm and 45 nm diameter NWs. 2-dimensional, (**e,f**), and 3-dimensional, (**g,h**), KPM maps of the surface potential of the NWs.

The NWs are [1,1,0] oriented, and have smooth surfaces with small random diameter variations as revealed by the scanning electron microscopy (SEM) and KPM topography images (Fig. 2a–d). Transmission electron microscopy (TEM) measurements reveal that the nanowires are single-crystalline. While the NWs bulk exhibits neither apparent structural defects nor observable strain effects, the surface contains vacancy defects and shows a slightly randomly fluctuating lattice constant. As shown in the two-dimensional plot of Fig. 2(e,f) as well as in the three-dimensional KPM potential maps, we observe equipotential lines along the NW perimeter and potential fluctuations along the nanowire axis. These potential ripples were observed for all the NWs with three different sizes. They have an average separation of 90 nm–200 nm, increasing with increasing NW diameter. The average peak-to-peak amplitudes is 12 mV–80 mV with no obvious dependence on NW diameter. These surface potential maps are independent of the scanning direction of the KPM and are reproducible with different AFM tips, thus excluding the possibility of experimental artifacts in the ripple structures. Moreover, the potential fluctuations are absent on Au and SiO_2_ surfaces in the same sample, confirming that these oscillations are an intrinsic property of the NWs instead of the contact materials or electrical noise. The average separation and amplitude of these potential ripples show no observable difference for the parts of the NW on top of Au electrodes and the freestanding parts. Moreover, these potential ripples were observed also in a nanowire on the gold layer without electrodes at its ends.

Such spatial surface potential fluctuations are one of the most significant types of disorder in the 2D surface states of 3D topological insulators when their Fermi level is close to the Dirac point^9^,^10^,^12^. They originate from modulations of the local density of states (LDOS), previously assigned to surface adsorbates, bulk impurities, structural defects, surface topology or charge disorder in the bulk. However, for our TI Bi_2_Te_3_ NWs no correlation was observed between SEM images and KPM topography images with the KPM potential images, and it is not possible to directly correlate the TEM images with the potential images since different NWs were imaged. In 3D topological surface states none of the structure/charge disorder effects is expected to induce modulations in the local chemical potential in a specific spatial direction, but rather non-directional random fluctuations across the whole 2D plane^37^.

Since the surface potential images correspond to the projections of the curved NW surfaces onto the substrate plane, we conclude the actual NW surface potential consists of equipotential rings around the nanowire perimeter, attributed to azimuthal 1D surface modes on the TI NWs surface as expected from theory^22^,^23^. Note that a previous paper^17^ reported Bi_2_Te_3_ nanowires with similar preparation methods (electrodeposition) and similar sizes as ours. Their magnetoconductance results confirms the presence of Dirac surface states, and therefore the Fermi level of their sample must lie near the Dirac point. We expect the same for our nanowires due to the similarity in composition, preparation method, size, and even device fabrication methods. The picture drawn from our experiments is that the azimuthal 1D surface states of TI NWs are relatively stable against a certain amount of distortion, but respond similarly to spatial disturbances in the local chemical potential along the NW axis as their 2D counterparts.

## Electrical conductivity

**Figure 3:**
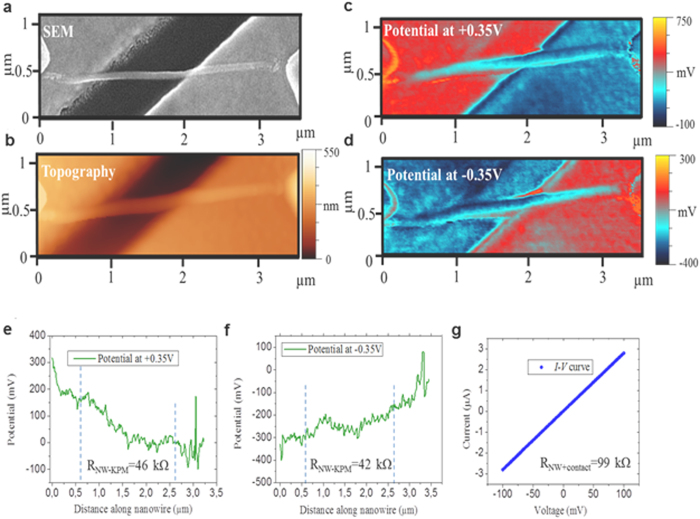
Accurate determination of electrical conductivity via surface potential mapping. (**a,b**) show SEM and AFM topographic images of a 45 nm diameter nanowire. (**c,d**) show the KPM 2D-surface potential map of the nanowire while applying a bias of 0.35 V and −0.35 V to the left electrode, respectively. (**e,f**) shows the surface potential profile along the nanowire for the two different bias applied. The dashed vertical blue lines mark the region of voltage drop along the nanowire used in the analysis. From the slope of the potential drop and the current flowing through the wire, the electrical resistance can be determined. (**g**) Shows the *I*–*V* curve obtained from two-probe measurements.

**Figure 4:**
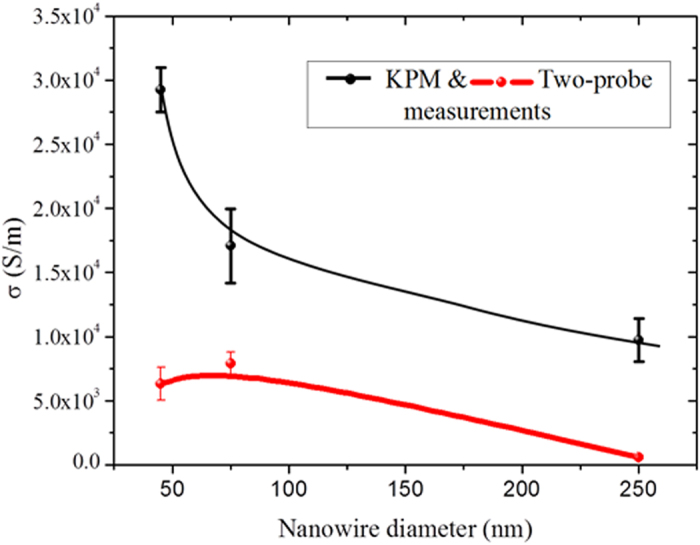
Electrical conductivity of nanowires with different diameter obtained with two-terminals *I*–*V* measurements and KPM methods. Lines are drawn to guide the eye.

**Figure 5:**
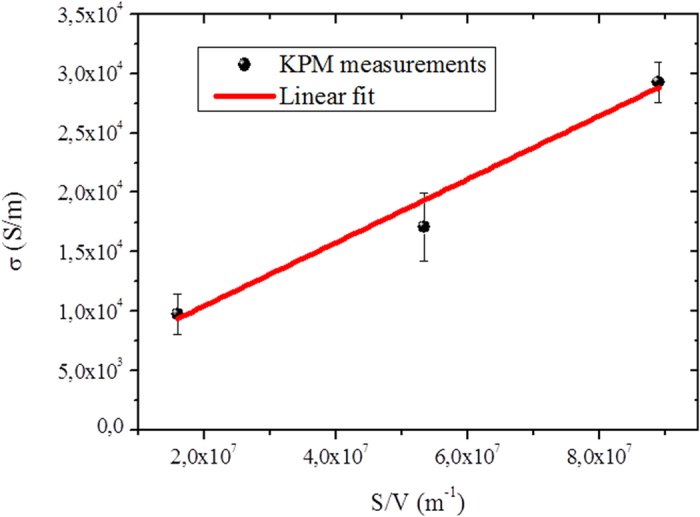
Electrical conductivity of nanowires obtained with KPM measurements versus surface to volume (S/V) ratio. Red line is a linear fit.

Now the question arises whether these surface potential ripples have an influence on the total electrical conductivity of the TI nanowires. From the discussion above it becomes clear that determining the actual electrical conductivity of a TI NW is not straightforward. The actual conduction pathways depends critically on the chemical potential and thus on the amplitude of the individual potential ripples^38^. To address this problem we proceed to map out the potential distribution of the NWs surface under a bias voltage applied across the NWs between the tungsten contacts. The results for a 45 nm NW are shown in Fig. 3. The electrode on the right was grounded, while a bias of ±0.35 V was applied to the left electrode. We observed that the potential changes roughly linearly along the NW (Fig. 3c–f), with the previously discussed periodic ripples superimposed on the potential change. We found that the average periodicity and amplitude of the ripples are independent of the bias (up to –0.75 – + 0.75V), indicating that the observed ripples are robust in real device operating conditions. From right to left, there is an increase of ~160 mV and a decrease of ~148 mV when applying 0.35 V and −0.35 V bias, respectively. Comparing the applied bias voltage to the change of potential along the NWs we found that in conventional two-terminal *I*–*V* measurements an error up to 50% can occur, due to the contact resistance. Two terminal *I*–*V* measurements on this NW give a total resistance of 99 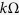 (Fig. 3g). From the potential profiles along the NW, we obtain an average contact resistance of 56 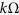 and consequently an actual NW resistances of 46 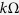 and 42 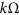, at 0.35 V and −0.35 V bias, respectively. Taking the NW diameter and length into account, we finally obtain an electrical conductivity *σ* of (2.93 ± 0.17) × 10^4^ S/m. Applying this method to the 75 nm and 250 nm NWs we obtain contact resistances of 18 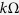 and 30 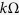, respectively. The resulting *σ* obtained from both methods (two-terminal and KPM) for all the NWs are shown in Fig. 4. We observe a clear diameter dependence of *σ,* which was accurately determined by KPM. It increases with decreasing NW diameter, as theoretically expected^39^ for TI NWs due to the increasing surface-to-volume ratio (*s*/*v*). Fitting the actual electrical conductivity vs. *s*/*v* (Fig. 5) to a simple two-channel model *σ* = *σ*_b_ + *σ*_s_(*s*/*v*), where the subscripts b and s refer to the bulk and surface, gives a bulk electrical conductivity of (0.51 ± 0.20) × 10^4^ S/m, which agrees well with literature values. Consequently, the surface contribution to total electrical conductivity of the nanowires is 43%–77%, increasing with decreasing diameter, demonstrating the significance of the TI surface states in room temperature NW devices. This size-dependent conductivity is otherwise obscure from simple *I*–*V* measurements. Despite the presence of potential ripples, surface state transport is still significant, likely due to the topological protection which makes it immune to backscattering. Our observation is consistent with previous magnetotransport measurements of Aharonov-Bohm (AB) oscillations and Subnikov-de Hass (SdH) oscillations of Bi_2_Te_3_ NWs, which revealed surface state dominated transport at low temperature^40^.

## Discussion and Conclusions

We have observed azimuthal equipotential rings on the surface of TI Bi_2_Te_3_ NWs of 45–250 nm diameter, rippled along the NW axis with peak-to-peak amplitudes of 12–100 mV and average separations of 90–215 nm. The azimuthal equipotential rings are assigned to 1D modes as expected for TI NWs from theory^3^. Along the NW axis these 1D modes behave similarly under perturbations, as reported for 2D Dirac systems like graphene^7^,^14^ and 3D TI surface states^37^,^41^. However, the origin of the surface potential ripples in our NWs cannot be clearly assigned to a single effect. Possible mechanisms include: (1) substrate-induced strain effects that lead to periodic structural rearrangements, as observed previously in strained VO_2_ nanobeams^42^ and suspended graphene sheets^43^; (2) surface chemical impurities as a result of the NW growth; (3) uneven surface oxidation; (4) unintentional and compositional heterogeneities in the nanowire bulk. To investigate the explicit role of the different mechanisms listed above, further in-depth studies are required. By taking the potential distribution as well as the contact resistance into account we showed that the surface contribution to the electrical conductivity in our Bi_2_Te_3_ NWs is up to 77%, increasing with decreasing diameter. Thus the surface states in TI NWs can not only dominate at low temperatures, but also play a significant role in their electrical transport at room temperature. Combining this high surface contribution with control over the structural and/or chemical disorder to modulate the NWs’ local potential energy landscape might pave the way to practical TI NW devices at room temperature for thermoelectric, spintronic, or quantum computing application^3^,^4^.

## Methods

### Preparation of the Bi_2_Te_3_ Nanowires

Bi_2_Te_3_ nanowires with different diameters were fabricated via pulsed electrodeposition inside of porous alumina templates with three different porous diameter^30^, ranging from 250 nm to 45 nm. A conventional three electrochemical cell and a bi-potentiostat were used. The porous alumina template is placed on the working electrode, the counter-electrode is a platinum wire and the reference electrode is Ag/AgCl. The solution used to obtain Bi_2_Te_3_ nanowires is described in ref. 44. In order to have dispersed nanowires in solution, the porous alumina was dissolved in a phosphoric acid (7 wt.%) and chromic oxide (1.8 wt.%) solution at 45 °C for one day. This solution was filtered with ethanol under vacuum conditions to pick up the nanowires.

### Structural characterization

The composition and crystalline orientation of the nanowires was carried out with a transmission electron microscopy (TEM) in the Molecular Foundry at the Lawrence Berkeley National Laboratory (LBNL) (Supplementary Information). X-Ray diffraction (XRD) and Scanning Electron Microscopy (SEM) were performed at the Servicio Interdepartamental de Investigación (SIdI) of the Universidad Autónoma de Madrid (UAM).

### Microchip fabrication

A Si wafer (1–10 Ω·cm) on which we deposited 300 nm of SiO_x_ by plasma-enhanced chemical vapor deposition (PECVD) was used to obtain an insulating substrate. Afterwards, we deposited 5 nm of chromium (Cr) and 200 nm of gold (Au). The focused ion beam (FIB) was used to draw a 1 μm gap coil/line that divides the deposited gold in two parts, each serving as an electrode. Gallium ions were used at an aperture of 60 μm, an intensity of 120 pA and a dose of 25.5 mC/cm^2^. In Supplementary Information, an optical image of the coil drawn by FIB is shown. Then, by drop cast, we deposited NWs on top of the microchip, and searched for single nanowires bridging the gap. In order to make good electrical contact to the nanowires, the end of the nanowires were cut and a local metal deposition (tungsten) was done through a gas injection system (GIS). This deposition was carried out at 3 ×10^−6^ mbar, a reservoir temperature for the tungsten of 60 °C, an aperture of 30 μm, current of 18 pA, a refreshing time of 0.015 ms and a dose of 400 mC/cm^2^.

### Kelvin Probe Microscopy

To resolve the surface potential (chemical potential) of the NWs and measure the electrical conductivity of the nanowires, we used a home-built KPM setup, which measures topography and surface potential distribution simultaneously, in a single-pass, frequency modulation mode.

## Additional Information

**How to cite this article**: Muñoz Rojo, M. *et al.* Spatial potential ripples of azimuthal surface modes in topological insulator Bi_2_Te_3_ nanowires. *Sci. Rep.* **6**, 19014; doi: 10.1038/srep19014 (2016).

## Supplementary Material

Supplementary Information
