# Supplementary material for: Spatial potential ripples of azimuthal surface modes in topological insulator Bi2Te3 nanowires
Source: Sci Rep. 2016 Jan 11;6:19014. doi: 10.1038/srep19014 (PMC4707462; doi:10.1038/srep19014)
Supplement: Supplementary Information [file srep19014-s1.doc]

Supplementary Information: Spatial potential ripples of azimuthal surface modes in topological insulator Bi_2_Te_3_ nanowires

**Miguel Muñoz Rojo^1^†, Yingjie Zhang^2,3^†, Cristina V. Manzano^1^, R. Alvaro^1^, Johannes Gooth,^4^ Miquel Salmeron^2^ and Marisol Martín González*^1^.**

† These authors contributed equally to this work.

*Corresponding Author: [marisol@csic.es](mailto:marisol@csic.es)

^1^ IMM-Instituto de Microelectrónica de Madrid (CNM-CSIC), Isaac Newton 8, PTM, E- 28760Tres Cantos, Madrid, Spain.

^2^ Materials Sciences Division, Lawrence Berkeley National Laboratory, 1 Cyclotron Road, Berkeley, CA 94720, United States.

^3^ Applied Science and Technology Graduate Program, University of California at Berkeley, Berkeley, CA 94720, USA.

^4^ Institute of Nanostructure and Solid State Physics, Universität Hamburg, Jungiusstrasse 11, 20355 Hamburg, Germany.

**S1. Nanowire Fabrication and Structural characterization.**

Bi_2_Te_3_ nanowires with different diameters were fabricated via pulsed-electrodeposition in similar conditions to ref. [^1^](#_ENREF_1) into porous alumina matrix. The fabrication of these templates were performed by a two-step anodization process, with which one can achieve alumina templates with pores diameters ranging from 45nm to 250nm [^2^](#_ENREF_2) [^3^](#_ENREF_3).

The electrodeposition was carried out in a conventional three electrode electrochemical cell using a bi-potentiostat. The porous alumina template is placed on the working electrode, the counter-electrode is a platinum wire and the reference electrode is Ag/AgCl. The solution used to obtain Bi_2_Te_3_ nanowires is described in ref. [^4^](#_ENREF_4). In order to have dispersed nanowires in solution, the porous alumina was dissolved in a phosphoric acid (7 wt. %) and chromic oxide (1.8 wt.%) solution at 45 ˚C for one day. This solution was filtered with ethanol under vacuum conditions to pick up the nanowires in a similar way to ref. [^5^](#_ENREF_5). Finally, the filter was immersed in a little flask with ethanol in order to have nanowires dispersed in solution. Figure S1a and Figure S1b show a transmission electron microscopy (TEM) of a 45 nm nanowire. In Figure S1a the diffraction pattern of the nanowire is also shown.


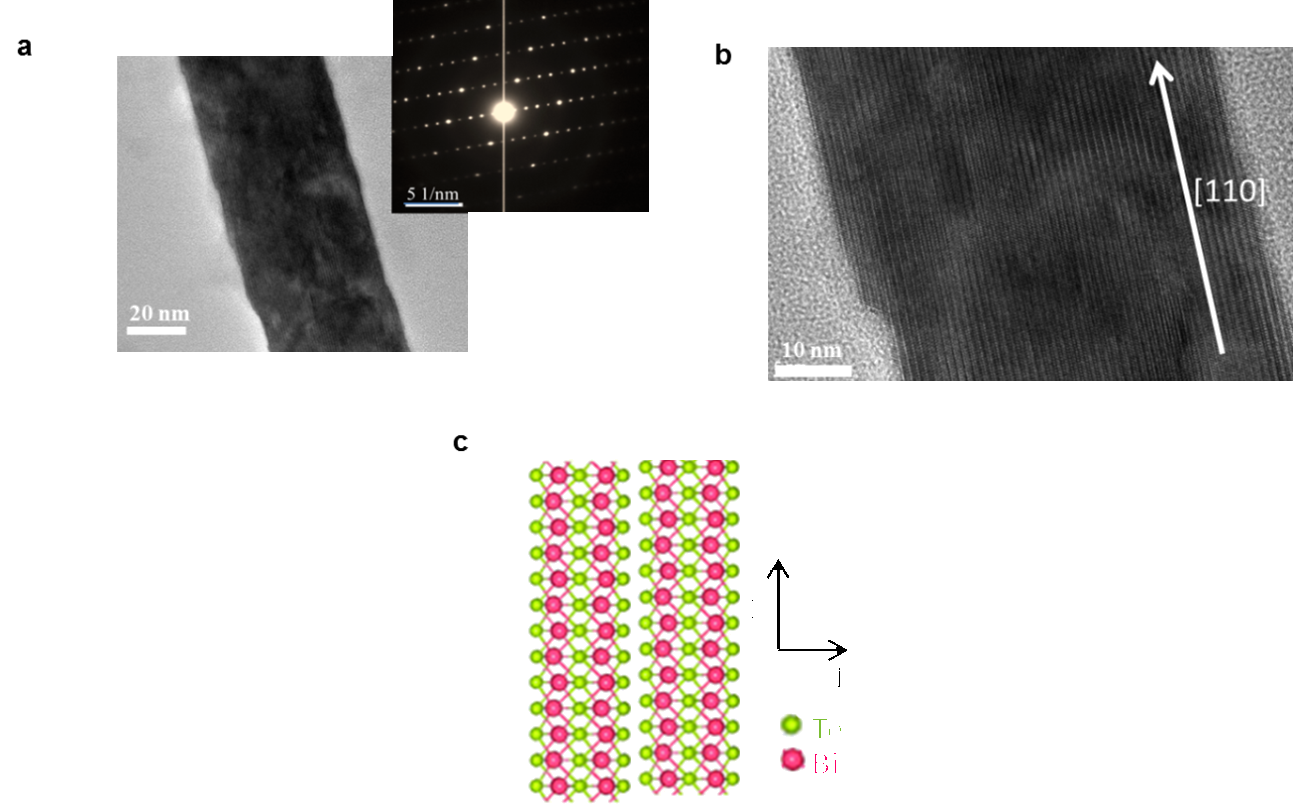


**Figure S1**. a) Transmission electron microscopy image of a 45 nm nanowire with its diffraction pattern. b) Zoom in of the nanowire that is oriented in the [110] direction. c) Schematic view of the crystalline structure of the Bi_2_Te_3_ atoms along the axis of the nanowire.

We grew n-type bismuth telluride nanowires with 250nm, 75nm and 45nm diameters. Their composition was evaluated by energy dispersive X-rays (EDX) and showed a ratio Bi-to-Te of 42% to 58%, with a variation of less than 5% between NWs with different diameters. The axis of the nanowire is preferentially oriented in the [110] crystallographic direction with a lattice distance of 3.68 Å, which was analyzed by TEM and shown schematically in Figure S1c.

**S2. Microchip fabrication.**

In order to study the electrical properties of nanowires, we fabricated microchips consisted of two electrodes separated by a gap. For that purpose, we used a Si wafer (1-10 Ω·cm) on which we deposited 300nm of SiO_x_ by plasma-enhanced chemical vapor deposition (PECVD) to obtain an isolating substrate. Afterwards, we deposited 5 nm of chromium (Cr) and 200nm of gold (Au). The focused ion beam (FIB) was used to draw a 1µm gap coil/line that divides the deposited gold in two parts, each serving as an electrode. Gallium ions were used at an aperture of 60 µm, an intensity of 120 pA and a dose of 25,5 mC/cm^2^. Figure S2a a shows an optical image of the coil drawn by FIB. Then, by drop cast, we deposited NWs on top of the microchip, and searched for singles nanowire bridging the gap. In order to make good electrical contact to the nanowire, the end of the nanowires were cut and a local metal deposition (tungsten) was done through gas injection system deposition (GIS). This deposition was carried out at 3·10^-6^ mbar, a reservoir temperature for the tungsten of 60 ºC, an aperture of 30 µm, current of 18 pA, a refreshing time of 0.015 ms and a dose of 400 mC/cm^2^. Figure S2b shows a scanning electron microscopy (SEM) image of a connected nanowire.


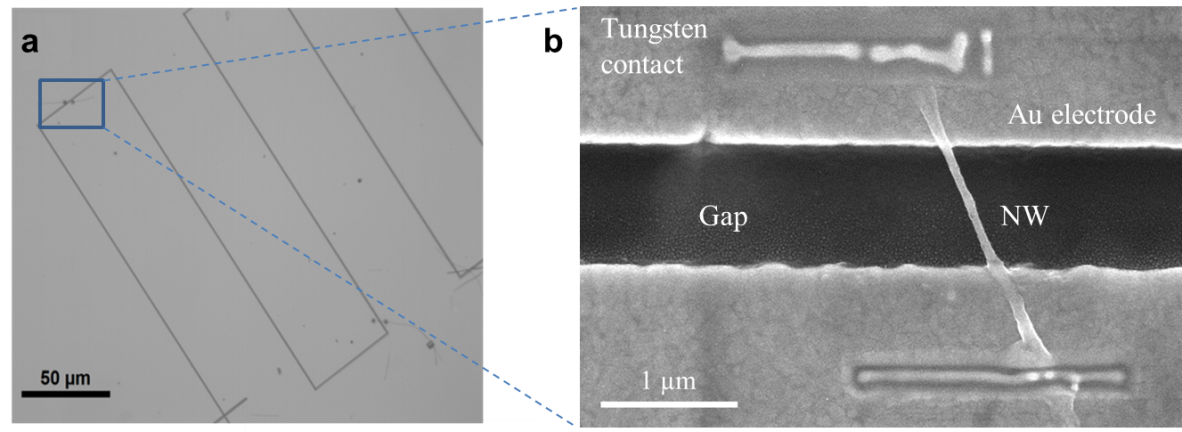


**Figure S2**. a) Optical image of the coil. b) SEM image of a 75 nm diameter nanowire bridging the gold electrodes with metal contacts.

**S3. Electrical conductivity measurements.**

Besides the NW shown in Figure 4, a couple more NWs were measured. As an example, Figure S3a shows a topographic image of a 250 nm nanowire, which bridges the 1µm gap between the gold electrodes. Figure S3b, S3c show the surface potential images of this nanowire with zero and 0.5 V bias applied across it, respectively. Figure S3d shows the potential profile of the NW under 0.5 V bias.


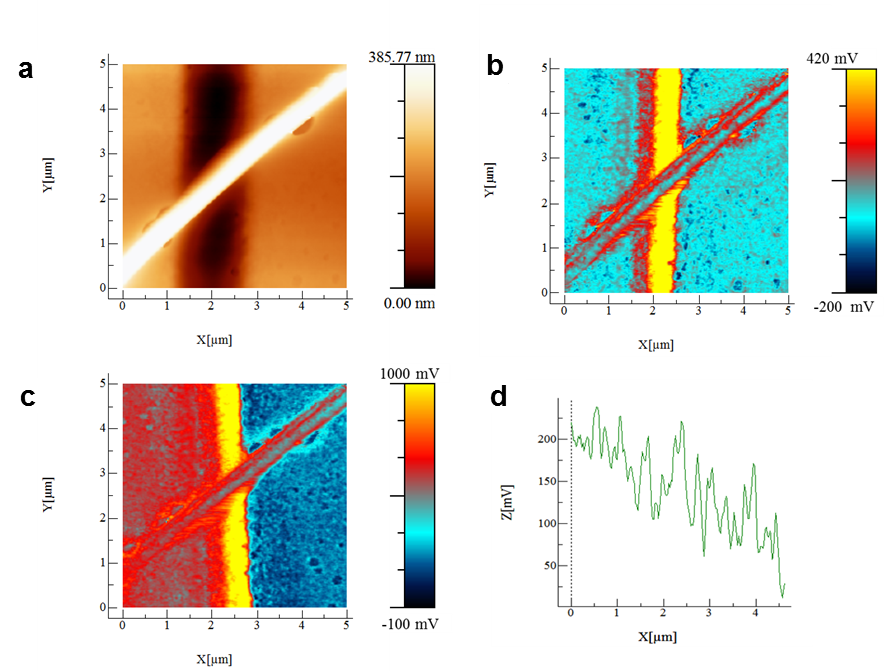


**Figure S3.** a) Topographic image of the 250 nm nanowire. Kelvin probe maps at b) 0V and c) 0.5V (to the left electrode). d) Profile of the linear voltage drop along the nanowire suspended in the gap part corresponding to the image in c).

In Figure S3b, under no voltage bias applied between electrodes, we observe that the surface potential of the Bi_2_Te_3_ nanowire is very similar to the one of the gold electrode. This is due to the fact that the work functions of both materials are similar, i.e. 5.3 eV for Bi_2_Te_3_ [^6^](#_ENREF_6) and 5.0-5.45 eV for gold [^7^](#_ENREF_7). Electrical conductivity of the NW is determined from the KPM images and I-V curves, using the following equation


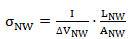
 (1)

where I and ΔV_NW_ are the current and potential drop along the nanowire, while L_NW_ and A_NW_ are the length and cross sectional area of the nanowire. The voltage drop measurements along the nanowire carries an uncertainty that was calculated from the standard deviation obtained from different voltage drop profiles analized from the NW. From this we obtain the standard error of electrical conductivity. The linear fit from the data in Figure 5 gives a bulk conductivity of (0.51±0.20)
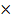
10^4^ S/m, where the error is from the standard error of the linear fit.

**S4. Grain boundaries/defects in nanowires revealed by surface potential mapping.**

As KPM can spatially map the local conductivity of a NW, we can microscopically understand the electrical transport mechanism that can be otherwise illusive when using 2-probe or 4-probe measurements alone. As an example, Figure S4 shows a NW where a drastic drop occurs near the middle of the nanowire when a voltage bias is applied across it, while AFM topography do not show clearly any visible defect and SEM only shows a slightly different contrast. The resistance of this NW is thus dominated by the defect which cannot be detected by two or four probe measurements and would result in a substantial underestimation of the electrical conductivity of the NWs using these techniques.


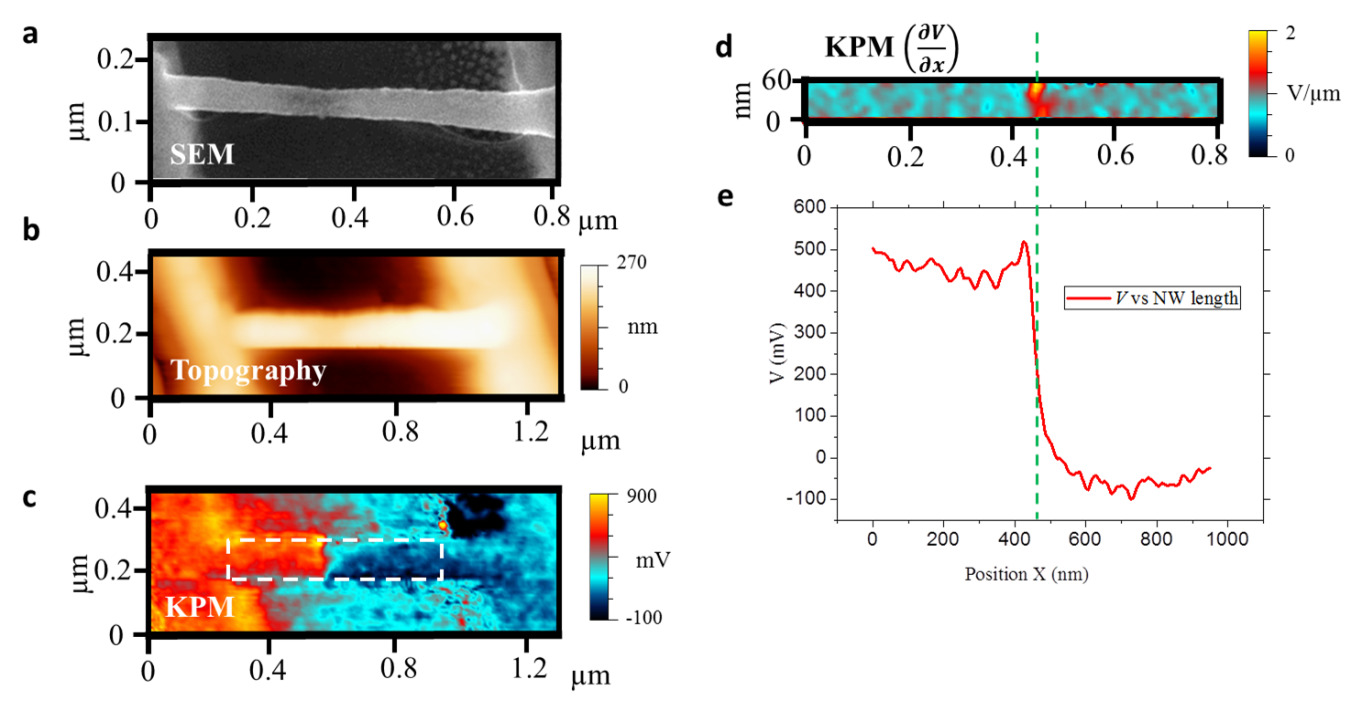


**Figure S4.** a) Topographic and b) SEM images of 75nm diameter nanowire. c) KPM map and d) derivative of the potential and e) the surface potential profile along the nanowire length (region inside the dashed rectangle) when a voltage bias of 0.5 V across it is applied to the left electrode.

**S5. Tungsten diffusion in the sample.**

In order to examine the possibility of metal diffusion on the NW, a KPM image was taken of the NW and the tungsten electrode to. The following figures S5a and S5b show the topographic and potential image of this tungsten-NW zone. In the topographic image (figure S5a), the blue square marks the tungsten contact in the nanowire, which has a size of around ~1 µm^2^. Figure b, shows that tungsten is slightly diffused intothe gold electrode while its diffusion to the nanowire is negligible. Moreover, the contacts were fabricated at a large distance away from the gap.


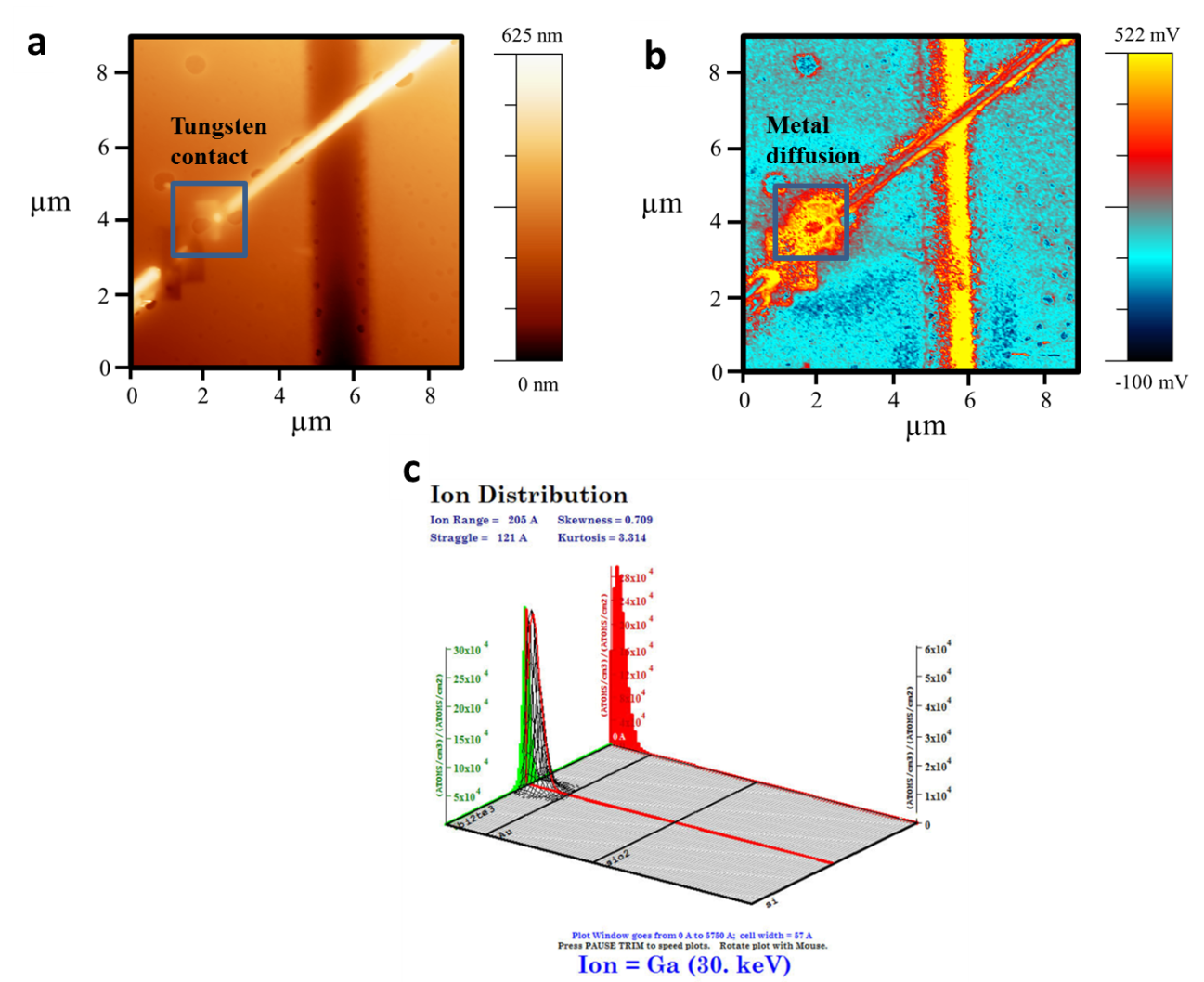


Figure S5. a) Topographic image of the tungsten contact on a 250 nm diameter nanowire. b) Potential image of the contact at 0 bias where is observed the diffusion of the metal to the electrode but practically nothing to the NW. c) Simulation of the implantation of gallium ions in a 75 nm diameter nanowire during tungsten deposition at an energy of 30 keV. The grey base of the 3D graph show the different materials presented along the cross section of the sample, i.e. Bi_2_Te_3_ NW, Au electrode and SiO_2_ substrate. The green peak corresponds to the lateral distribution (later straggling) and the red is the penetration depth of the ions, while the black distribution is a combination of them.

Furthermore, figure c shows a simulation of the implantation of gallium ions at 30 keV for a 75 nm diameter NW during the tungsten deposition, according to the experimental conditions. The diffusion of gallium ions in the NWs was calculated to be around 12 nm, and is thus essentially negligible.

**References**

1 Manzano, C. *et al.* Thermoelectric properties of Bi_2_Te_3_ films by constant and pulsed electrodeposition. *J. Solid State Electrochem.* **17**, 2071-2078 (2013).

2 Prieto, A. *et al.* Electrodeposition of ordered Bi_2_Te_3_ nanowire arrays. *J. Am. Che. Soc.* **123**, 7160-7161 (2001).

3 Martín, J., Manzano, C. & Martín-González, M. In-depth study of self-ordered porous alumina in the 140–400 nm pore diameter range. *Microporous and Mesoporous Mater.* **151**, 311-316 (2012).

4 Martin Gonzalez, M., Prieto, A., Gronsky, R., Sands, T. & Stacy, A. Insights into the electrodeposition of Bi_2_Te_3_. *J. Electrochem. Soc.* **149**, C546-C554 (2002).

5 Vega, V. *et al.* Tuning the magnetic anisotropy of Co-Ni nanowires: comparison between single nanowires and nanowire arrays in hard-anodic aluminum oxide membranes. *Nanotechnology* **23**, 465709 (2012).

6 Haneman, D. Photoelectric emission and work functions of InSb, GaAs, Bi_2_Te_3_ and germanium. *J. Phys. Chem. Solids* **11**, 205-214 (1959).

7 Sachtler, W., Dorgelo, G. & Holscher, A. The work function of gold. *Surf. Sci.* **5**, 221-229 (1966).
